# Ventilator-associated pneumonia is linked to a worse prognosis than community-acquired pneumonia in children

**DOI:** 10.1371/journal.pone.0271450

**Published:** 2022-07-14

**Authors:** Maria Hernandez-Garcia, Monica Girona-Alarcon, Sara Bobillo-Perez, Mireia Urrea-Ayala, Anna Sole-Ribalta, Mònica Balaguer, Francisco-José Cambra, Iolanda Jordan

**Affiliations:** 1 Paediatrics Department, Hospital Sant Joan de Déu, University of Barcelona, Barcelona, Spain; 2 Paediatric Intensive Care Unit, Hospital Sant Joan de Déu, University of Barcelona, Barcelona, Spain; 3 Immunological and Respiratory Disorders in the Paediatric Critical Patient Research Group, Institut de Recerca Hospital Sant Joan de Déu, Hospital Sant Joan de Déu, Barcelona, Spain; 4 Emergency Transport System, Hospital Sant Joan de Déu, Barcelona, Spain; 5 Infection Control Unit, Hospital Sant Joan de Déu, University of Barcelona, Barcelona, Spain; 6 Paediatric Intensive Care Unit, CIBERESP, Hospital Sant Joan de Déu, University of Barcelona, Barcelona, Spain; LSU Health Shreveport, UNITED STATES

## Abstract

**Background:**

Around 12–20% of patients with community-acquired pneumonia (CAP) require critical care. Ventilator-associated pneumonia (VAP) is the second cause of nosocomial infection in Paediatric Intensive Care Units (PICU). As far as we know, there are no studies comparing both types of pneumonia in children, thus it remains unclear if there are differences between them in terms of severity and outcomes.

**Objective:**

The aim was to compare clinical and microbiological characteristics and outcomes of patients with severe CAP and VAP.

**Methods:**

A retrospective descriptive study, including patients diagnosed of VAP and CAP, with a positive respiratory culture and under mechanical ventilation, admitted to the PICU from 2015 to 2019.

**Results:**

238 patients were included; 163 (68.4%) with CAP, and 75 (31.5%) with VAP. Patients with VAP needed longer mechanical ventilation (14 vs. 7 days, *p*<0.001) and more inotropic support (49.3 vs. 30.7%, *p* = 0.006). Patients with VAP had higher mortality (12 vs. 2.5%, *p* = 0.005).

*Enterobacterales* were more involved with VAP than with CAP (48 vs. 9%, *p*<0.001). Taking into account only the non-drug sensitive microorganisms, patients with VAP tended to have more multidrug-resistant bacteria (30 vs. 10.8%, *p* = 0.141) than patients with CAP.

**Conclusion:**

Patients with VAP had worse prognosis than patients with CAP, needing longer mechanical ventilation, more inotropic support and had higher mortality. Patients with VAP were mainly infected by *Enterobacterales* and had more multidrug resistant microorganisms than patients with CAP.

## Introduction

Pneumonia is one of the most common causes of infection requiring hospitalisation in children, and it is the most frequent reason for antibiotic use in paediatric hospitals [1,2]. Pneumonia in children can be classified as either community-acquired pneumonia (CAP) or ventilator-associated pneumonia (VAP).

CAP is currently one of the primary causes of mortality in children worldwide, especially in those under 5 years old [3,4]. Although mortality is lower in developed countries, CAP is still associated with substantial morbidity and remains the most common indication for paediatric hospitalisation outside the newborn period in the United States [1].

Moreover, about 12–20% of paediatric CAP cases require critical care [1,5,6], primarily due to the development of respiratory failure requiring assisted ventilation and pneumonia complicated by septicaemia [3]. Risk factors that contribute to developing severe CAP are the presence of underlying comorbidities, including prematurity, bronchopulmonary dysplasia, congenital heart disease, immunodeficiency, and severe cerebral palsy. Another risk factor is a relevant medical history of severe pneumonia [3,7].

Among the different hospital-acquired infections in children, VAP is the most common after blood stream infections. In paediatric intensive care units (PICUs), patients on mechanical ventilation (MV) run the risk of developing VAP, which is estimated to occur in around 10–20% of ventilated patients. As is the case with CAP, VAP involves high morbidity and mortality; it can prolong the length of respiratory support and hospitalisation, increase the mortality rate, and directly affect patient outcomes in PICUs [6,8,9]. Age less than 1 year, altered immune status, unplanned emergency intubations and reintubation, acute respiratory distress syndrome, continuous enteral feeding and use of discontinuous sedation have been associated with an increased risk of developing VAP [8–10].

Severe CAP requiring admission to the PICU and VAP differ not only from an aetiological and pathophysiological point of view, but also as regards their microbiological findings. Identifying the most common bacterial pathogens is important to aid in decisions related to empirical antibiotics [11].

It is known that the bacterial pathogens most frequently related to CAP are gram positive bacteria and non- *Enterobacterales* gram negative bacteria, especially *S*. *pneumoniae*, *S*. *aureus*, *H*. *influenzae* and *M*. *catarrhalis* [12,13]. By contrast, the most frequent bacterial pathogens isolated in VAP are *Enterobacterales* and other gram negative bacteria [9]. Regarding the susceptibility pattern, it has been reported that multidrug and extensively drug-resistant microorganisms are more common among patients with VAP [14]. Due to this, dealing with VAP in any intensive care unit is challenging. It is important to identify the burden of VAP in any setting, so that prevention strategies can be implemented and strengthened.

As far as we know, there are no published references in paediatrics comparing VAP and CAP in terms of the risk factors, the microorganisms related to them, and the outcomes. Our hypothesis was that CAP and VAP in critically ill children could not have the same severity. Therefore, we decided to analyse the differences between them in a paediatric intensive care unit (PICU). The main objective of the study was to describe the clinical and microbiological characteristics of severe CAP and VAP. The secondary objective was to compare the different outcomes depending on the type of pneumonia.

## Material and methods

This was a single-centre retrospective and observational study performed in the PICU of Hospital Sant Joan de Déu in Barcelona, which is a tertiary referral paediatric hospital with 326 beds and approximately 1,200 PICU admissions per year.

Patients under 18 years old admitted to the PICU from January 2015 to December 2019 who required MV with a confirmed diagnosis of VAP or CAP were included. Patients without a positive tracheal aspirate culture or those with < 10^5^ colonies/mL were excluded, in order to exclude colonisations and contaminations.

Patients were classified depending on their diagnosis, according to following:

- **CAP:** According to Shah *et al*. [15], presence of signs and symptoms of pneumonia in a previously healthy child caused by an infection that has been acquired outside of the hospital. Signs and symptoms of pneumonia include clinical data (fever ≥ 38°C, tachypnoea, nasal flaring, grunting, retractions, hypoxia), chest X-ray opacities, and increased levels of acute phase reactants (CRP ≥ 70mg/dL and/or PCT ≥ 1ng/mL) [16–18]. Patients needed to fulfill all 3 diagnostic criteria. A microbial diagnosis required a positive respiratory culture and/or a positive blood culture and/or a positive PCR for *S*. *pneumoniae* or *S*. *aureus*.
- **VAP:** According to the CDC criteria [19], defined as a pneumonia where the patient is on mechanical ventilation for at least 2 calendar days on the date of diagnosis, with the day they were placed on the ventilator being day 1 AND the ventilator being in place on the date of diagnosis or the day before. Diagnosing VAP requires clinical data (worsening respiratory assessment), radiological findings (consolidations on X-ray or worsening thereof), and a positive culture (tracheal aspirate, ≥ 10^5^ CFU/mL) [11,20,21].

The study was approved by the local Clinical Research Ethics Committee (PIC-180-19), and it was carried out in compliance with the Declaration of Helsinki.

### Variables

The following demographic data were collected from the electronic medical records: age, gender, comorbidities, Paediatric Risk of Mortality Score (PRISM III) [22] at admission, and reason for admission (respiratory, cardiovascular, neurologic, haemato-oncological, surgical, sepsis). Risk factors: days in the hospital until intubation, days in the hospital until infection, and days on MV until infection. The antibiotic therapy used was recorded (including duration and the need to switch medications). The microbiological data collected included the microorganisms isolated in the blood culture and respiratory cultures and their antibiotic susceptibility (defined according to European Centre for Disease Prevention and Control criteria) [20]. As for the latter measure, bacteria were classified as either drug-susceptible (DS) or non-drug-susceptible (non-DS). The non-DS group included drug resistant (DR, if non-susceptible to at least 1 agent in less than 3 categories) and multidrug-resistant (MDR, if non-susceptible to at least 1 agent in 3 or more categories) [23]. Specific resistance phenotypes, such as extended-spectrum beta-lactamase (ESBL) and MRSA were also recorded. Outcomes were considered as the support required during the PICU admission, including the duration of the respiratory support (days on MV or non-invasive mechanical ventilation), the inotropic support in the PICU (requirement, duration, and the maximum vasoactive-inotropic score, (VIS), which is a widely used inotrope scoring system that includes dopamine, dobutamine, epinephrine and milrinone doses, and its punctuation is related to “poor outcome” [24,25], the need for extracorporeal support, the length of stay in the PICU and in the hospital, and the mortality during the their stay in the PICU. The presence of sepsis, defined according to Goldstein definition (the presence of at least two of the following criteria: temperature>38.5°C or <36°C, tachycardia, mean respiratory rate>2 SD above normal for age, leukocyte count elevated or depressed PLUS suspected or proven infection [26,27]) was also analysed in both groups (VAP and CAP). The analytical biomarkers recorded were C-reactive protein and procalcitonin, analysing the highest value of each one.

### Outcomes

The primary outcomes were to analyse demographical characteristics of patients with VAP and CAP, and to describe the microbiological data of severe CAP and VAP. Secondary outcomes were to analyse the differences in the respiratory and haemodynamic support between patients with CAP and VAP, the differences in hospital length of stay, in extracorporeal support, and mortality.

### Statistical analysis

The statistical analysis was performed using SPSS 25.0 Statistics®. Categorical variables were indicated as frequency (n) and percentage (%), whereas continuous variables were summarised as median and interquartile range (IQR) because they were not normally distributed. The comparison of categorical variables was performed using the χ2–test or Fisher’s exact test. Continuous variables were compared with the Mann-Whitney U test. Probability values of less than 0.05 were considered statistically significant.

Two multivariate analyses were performed: first one, to detect independent risk factors for VAP and, second one, to detect independent risk factors for mortality. In both cases, variables that were significant in the univariate analysis were entered into multiple forward stepwise logic regression models. Continuous variables were converted into dichotomous variables using Receiver Operating Characteristic curves to detect the best cut-off point (taking into account the sensitivity (Sn) and specificity (Sp)) for each one with respect to the dependent variable. The final models were those with the highest Hosmer and Lemeshow goodness-of-fit test result. These results were represented as Odds ratio (OR) and its 95% confidence interval (CI).

## Results

### Clinical characteristics

In total, 238 patients were included; 125 (52.5%) were males and the median age was 6.3 months (IQR 1.6–43.5). A total of 100 patients (42%) had some comorbidity. Among all the patients, 163 (68.4%) were diagnosed with CAP and 75 (31.5%) with VAP. The main demographical data are detailed in Table 1.

**Table 1:** Patient characteristics, risk factors, and outcomes.

	General (n = 238)	CAP (n = 163)	VAP (n = 75)	*p*
**Males, n (%)**	125 (52.5)	81 (49.7)	44 (58.7)	0.211
**Age (months), median (IQR)**	6.6 (1.6–46.4)	6.2 (1.4–51.2)	6.9 (1.8–28)	0.870
**Risk factors, n (%)** Comorbidity Tracheostomy	100 (42) 8 (3.4)	52 (31.9) 2 (1.2)	48 (64) 6 (8)	**<0.001** **0.013**
**Reason for admission, n (%)** Respiratory Cardiovascular Neurologic Surgical Sepsis Oncological Other	150 (63) 23 (9.7) 23 (9.7) 8 (3.4) 10 (4.2) 1 (0.4) 23 (9.7)	115 (70.6) 8 (4.9) 14 (8.6) 6 (3.7) 7 (4.3) 1 (0.6) 12 (7.4)	35 (46.7) 15 (20) 9 (12) 2 (2.7) 3 (4) 0 (0) 11 (14.7)	**0.002** **<0.001** **<0.001** 0.408 1.000 1.000 1.000 0.076
**PRISM III, median (IQR)**	4 (2–7)	4 (2–7)	5 (2–9)	0.079
**Risk factors until infection, median (IQR)**				
Days from admission to intubation	0 (0–0)	0 (0–0)	0 (0–2)	**0.002**
Days from admission to infection	3 (1–8)	1 (0–3)	11 (7–14)	**<0.001**
Days from intubation to infection	2 (0–8)	1 (0–3)	8 (6.5–12)	**<0.001**
**Biomarkers, median (IQR)**				
C-reactive protein (mg/L)	89 (45–159)	86.6 (40–152)	103 (54–167)	0.243
Procalcitonin (ng/mL)	1.35 (0.35–6.1)	1.3 (0.4–5.7)	1.7 (0.3–7.9)	0.850
Lactate (mmol/L)	2.3 (1.8–3)	2.2 (1.8–2.9)	2.4 (2–3)	0.191
**Treatment** Inotropic treatment, n (%) Inotropic length (days), median (IQR) Maximum VIS, median (IQR) Antibiotic length (days), median (IQR) Appropiate initial antibiotic, n(%) Antibiotic switched, n (%)	87 (36.6) 5 (2–9) 10 (5.5–29.5) 7 (7–10) 208 (87.4) 121 (50.8)	50 (30.7) 3 (2–5) 10 (5–28) 7 (7–8) 143 (87.7) 74 (45.7)	37 (49.3) 8 (4–13) 15 (10–30) 10 (7–13) 65 (86.7) 47 (63.5)	**0.006** **<0.001** 0.073 **<0.001** 0.818 **0.011**
**Outcomes** Days on MV, median (IQR) Days on NIV, median (IQR) Length of stay in ICU (days), median (IQR) Length of stay in hospital (days) Sepsis, n (%) Septic shock, n (%) Positive blood culture, n (%) ECMO needed, n (%) Mortality, n (%)	9 (6–14) 2 (0–5) 15 (9–22) 22 (15–34) 42 (17.6) 25 (59.5) 31 (13) 11 (4.6) 13 (5.5)	7 (4.7–12) 2 (0–4) 12 (7–16) 19 (14–26) 24 (14.7) 17 (70.8) 18 (11) 3 (1.8) 4 (2.5)	14 (11–21) 3 (1–8) 24 (18–38) 35.5 (26–57.3) 18 (24) 8 (44.4) 13 (17.3) 8 (10.7) 9 (12)	**<0.001** **<0.001** **<0.001** **<0.001** 0.081 0.085 0.180 **0.005** **0.005**

Values are expressed as frequency (percentage) for qualitative variables and compared using Chi-square test. Quantitative variables expressed as median (interquartile range) and compared using Mann-Whitney test. PRISM III: Paediatric Risk Mortality III score. CAP: Community-acquired pneumonia. VAP: Ventilator-associated pneumonia. VIS: Vasoactive Inotropic Score. MV: Mechanical ventilation. NIV: Non-invasive mechanical ventilation. ICU: Intensive care unit. ECMO: Extracorporeal membrane oxygenation.

Comparing patients with CAP vs. those with VAP, no differences were found as regards gender or age. Patients with VAP had a higher percentage of associated comorbidity (64% vs. 31.9%, *p*<0.001), and they were also more likely to have tracheostomies (8% vs. 2%, *p* = 0.013). Admission due to respiratory problems was more frequent in patients with CAP than in patients with VAP (70.6% vs. 46.7%, *p =* 0.001). Patients who developed VAP were admitted due to cardiovascular reasons more frequently than patients with CAP (20% vs. 4.9%, *p*<0.001).

While the diagnosis of CAP was made at day 1 (IQR 0–3), VAP was diagnosed at day 11 (IQR 7–14), p = 0.001. This difference was also significant when considering the days elapsed from the endotracheal intubation to the infection (1 vs. 8 days, *p* = 0.001).

No differences were found in C-reactive protein values (103 vs. 86.6 mg/L, *p* = 0.243) nor in procalcitonin levels (1.7 vs. 2.2 ng/mL, *p* = 0.191). Patients with VAP required longer antibiotic treatment (10 vs. 7 days, *p*<0.001) and more antibiotic switching (63.5% vs. 45.7%, *p* = 0.011).

Patients with VAP had a higher proportion of sepsis than patients with CAP (24% vs. 14.7%), even though the difference was not statistically significant (*p* = 0.081).

As regards the microbiological data, *Enterobacterales* were the microorganisms most frequently associated with VAP (n = 36, 48%), yielding a higher percentage than those patients with CAP (n = 5.5, 9%, *p*<0.001). The main microorganisms isolated in CAP were gram negative bacteria (n = 94, 57.7%), followed by gram positive bacteria (n = 60, 36.8%), both in higher proportions than in VAP (*p* = 0.05 and *p*<0.001, respectively). Looking at the specific microorganisms, CAP was more frequently related to *Haemophilus spp*. (*p*<0.001), *S*. *pneumoniae* (*p*<0.001), *S*. *aureus* (*p* = 0.021), and *M*. *catarrhalis* (*p* = 0.010) than VAP. VAP was more likely to be associated with *P*. *aeruginosa* (*p*<0.001), *Klebsiella spp*. (*p*<0.001), *Enterobacter spp*. (*p*<0.001), *E*. *coli (p* = 0.030), *S*. *maltophilia* (*p* = 0.003), and *Serratia spp* (*p* = 0.009) than CAP.

No differences were found in the percentage of DS microorganisms between groups (*p* = 0.505). Taking into account only the non-DS microorganisms, patients with CAP tended to have more DR bacteria (86.5% vs. 65%, *p* = 0.058) while patients with VAP tended to have more MDR (30% vs. 10.8%, *p* = 0.141) and ESBL (30% vs. 8.1%, *p* = 0.054) ones, even though the differences were not statistically significant. The main microbiological data are detailed in Table 2.

**Table 2:** Microbiological data of the respiratory samples.

		CAP (n = 163)	VAP (n = 75)	*p*
**Respiratory cultures** Type of microorganism • Gram positive • Gram negative *Enterobacterales* Resistance pattern • DS • Non-DS • DR • MDR • XDR • MRSA • ESBL		60 (36.8) 94 (57.7) 9 (5.5) 126 (77.3) 37 (22.6) 32 (86.5) 4 (10.8) 1 (2.7) 4 (10.8) 3 (8.1)	6 (8) 33 (44) 36 (48) 55 (73.3) 20 (26.6) 13 (65) 6 (30) 1 (5) 2 (10) 6 (30)	**<0.001** 0.05 **<0.001** 0.505 0.058 0.141 1.000 1.000 **0.054**
**Specific microorganisms**				
**Gram positive**	*Enterococcus spp*. *S*. *aureus* *S*. *pneumoniae* *S*. *pyogenes* *S*. *viridans*	1 (0.6) 27 (16.6) 28 (17.2) 3 (1.8) 1 (0.6)	1 (1.3) 4 (5.3) 0 (0) 0 (0) 1 (1.3)	0.532 **0.021** **<0.001** 0.554 0.532
**Gram negative**	*A*. *xylosoxidans* *B*. *cepacia* *Haemophilus spp*. *M*. *catarrhalis* *N*. *meningitidis* *P*. *aeruginosa* *P*. *mirabilis* *S*. *maltophilia*	0 (0) 0 (0) 66 (40.5) 22 (13.5) 1 (0.6) 5 (3.1) 0 (0) 0 (0)	1 (1.3) 2 (2.7) 5 (6.7) 2 (2.7) 0 (0) 17 (22.7) 1 (1.3) 5 (6.7)	0.315 0.098 **<0.001** **0.010** 1.000 **<0.001** 0.315 **0.003**
***Enterobacterales***	*Enterobacter spp*. *E*. *coli* *Klebsiella spp*. *M*. *morgannii* *Serratia spp*.	2 (1.2) 3 (1.8) 4 (2.5) 0 (0) 0 (0)	12 (16) 6 (8) 13 (17.3) 1 (1.3) 4 (5.3)	**<0.001** **0.030** **<0.001** 0.315 **0.009**

Values are expressed as frequency (percentage) for qualitative variables, and compared using the Chi-square test. CAP: Community-acquired pneumonia. VAP: Ventilator-associated pneumonia. DS: Drug-sensitive. Non-DS: Non-drug-sensitive. DR: Drug-resistant. MDR: Multidrug-resistant. XDR: Extensively drug-resistant. MRSA: Methicillin-resistant *staphylococcus aureus*. ESBL: Extended spectrum beta-lactamases.

**Fig 1:**
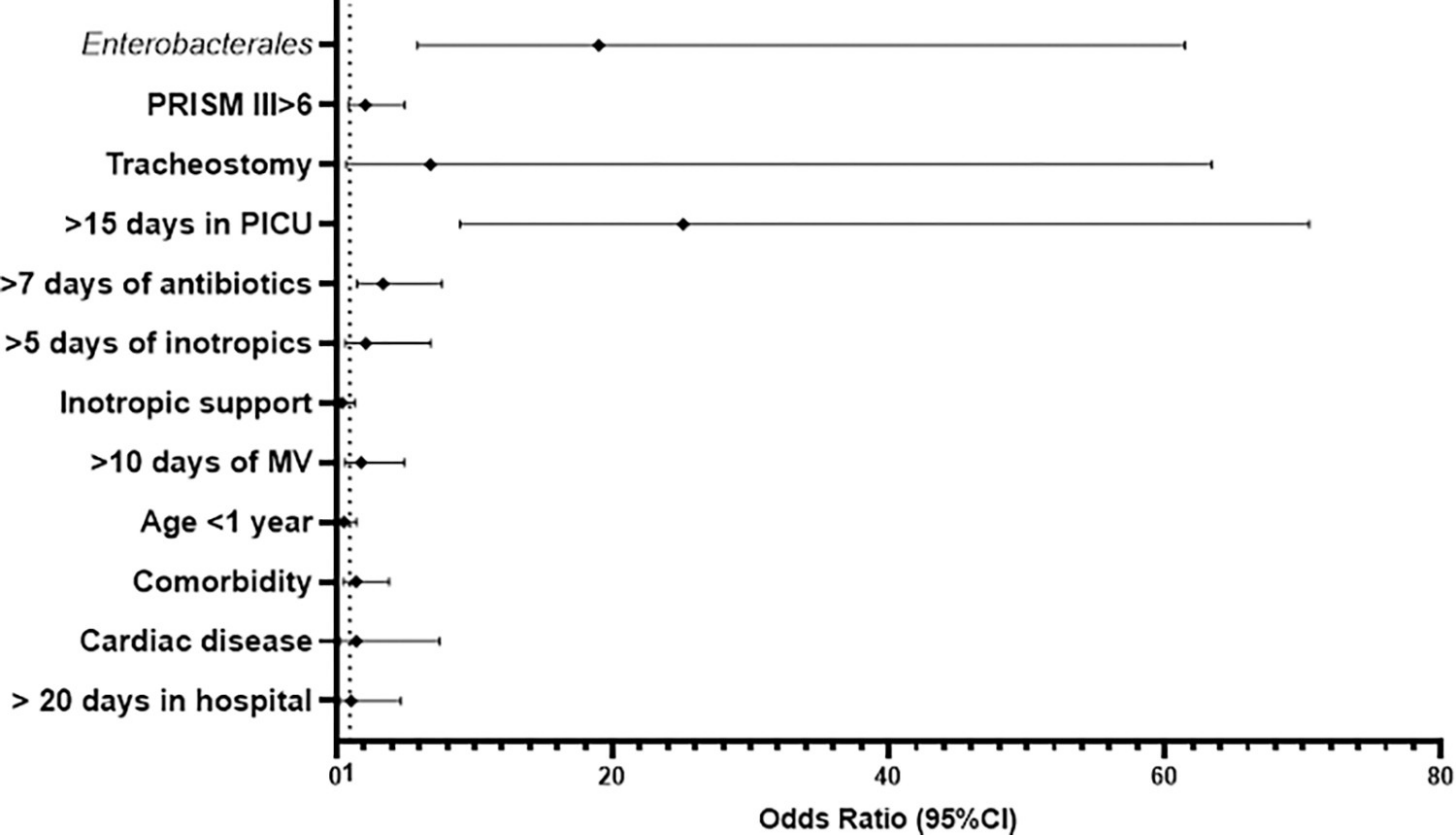
Forest plot representing the multivariate model to detect independent risk factors for ventilator-associated pneumonia. Cut-off points for continuous variables determined using Receiver Operating Characteristic curves: Antibiotic length: 7 days (Sn 72.5%, Sp 71.0%); Inotropic length: 5 days (Sn 75.7%, Sp 59.5%); days on mechanical ventilation (MV): 10 (Sn 84.1%, Sp 73.1%); Length of stay in PICU (paediatric intensive care unit): 15 days (Sn 88.4%, Sp 74.8%); Length of stay in hospital: 20 days (Sn 90.0%, Sp 59.1%).

A multivariate analysis was performed to detect independent risk factors for VAP, represented in Fig 1. After entering the significant variables found in the univariate analysis, according to the logistic stepwise regression the independent risk factors for VAP were: length of stay in PICU>15 days (OR 25.11, 95% CI 8.95–70.45), *Enterobacterales* (OR 19.01, 95% CI 5.88–61.46, *p*<0.001) and antibiotic treatment longer than 7 days (OR 3.40, 95% CI 1.51–7.67, *p* = 0.003).

### Outcomes

Patients with VAP required prolonged respiratory support: they needed more days of MV (14 vs. 7 days on MV, *p*<0.001), and non-invasive ventilation (3 vs. 2 days, *p*<0.001).

Patients with VAP needed inotropic support more often than patients with CAP (49.3% vs. 30.7%, *p* = 0.006) and for a longer period (8 vs. 3 days, *p*<0.001). They tended to require higher levels of inotropic treatment when comparing VIS score (15 vs. 10, *p* = 0.073), even though the difference was not statistically significant. Table 1 summarizes the main outcomes for the two groups.

Patients with VAP had a longer stay in the PICU (24 vs. 12 days, *p*<0.001) and in the hospital (35.5 vs. 19 days, *p*<0.001) than patients with CAP. They had a more frequent need for extracorporeal support (10.7% vs. 1.8%, *p* = 0.005) and had higher mortality rate (12% vs. 2.5%, *p* = 0.005).

**Fig 2:**
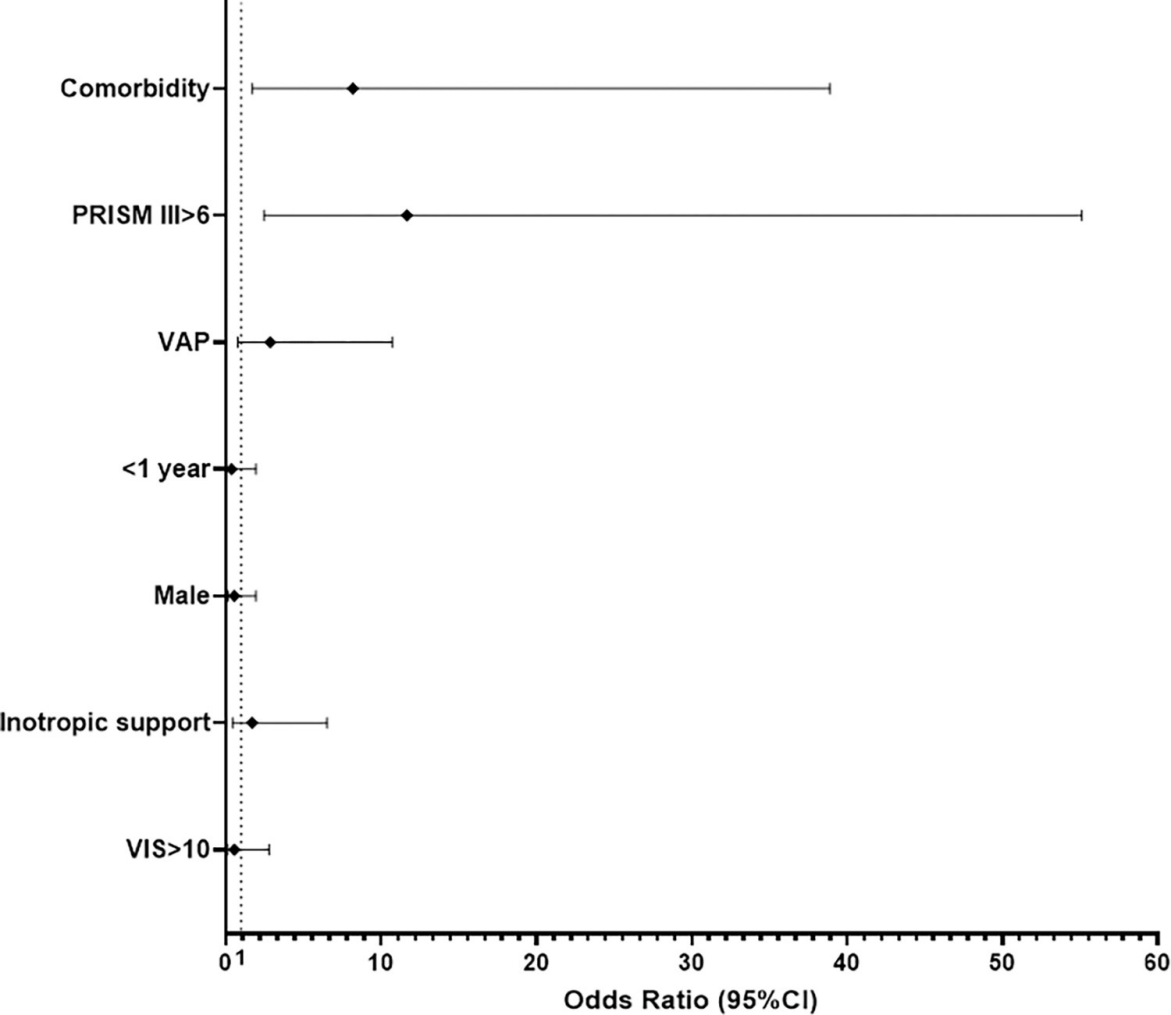
Forest plot representing the variables introduced in the multivariate model to detect independent risk factors for mortality. Cut-off points for continuous variables determined using Receiver Operating Characteristic curves: Paediatric risk of mortality score (PRISM III)>6 (Sn 84.6%, Sp 69.4%), Vasoactive inotropic score (VIS) (Sn 88.9%, Sp 34.2%).

Mortality was analysed separately, in order to detect if VAP was an independent risk factor for mortality or not. First, a univariate analysis was performed to detect risk factors for mortality, and the main risk factors for death were: age <1 year (OR 0.19, *p* = 0.021), comorbidity (OR 8.40, *p* = 0.001), PRISM-III>6 (OR 11.93, *p*<0.001), VAP (OR 5.42, *p* = 0.003), inotropic treatment (OR 4.24, *p* = 0.012) and VIS>10 (OR 3.08, *p* = 0.049), detailed in Table 3. Second, these significant risk factors were entered into a multivariate model, and the independent risk factors for death were PRISM III>6 (OR 11.66, 95% CI 2.47–55.11, *p* = 0.002) and the presence of comorbidity (OR 8.18, 95% CI 1.72–38.89, *p* = 0.008) (Fig 2).

**Table 3:** Univariate analysis of the risk factors of mortality.

Risk factors	Survivors n = 225	Exitus n = 13	*p*	OR (95%CI)
Male, n (%)	119 (52.9)	6 (46.2%)	0.636	0.764 (0.25–2.34)
Age <1year, n(%)	111 (49.3)	2 (15.4)	**0.021**	0.187 (0.04–0.862)
Comorbidity, n(%)	89 (39.6)	11 (84.6)	**0.001**	8.40 (1.82–38.82)
Tracheostomy, n(%)	7 (3.1)	1 (7.7)	0.366	2.60 (0.30–22.83)
Cardiovascular reason for admission, n(%)	21 (9.3)	2 (15.4)	0.364	1.77 (0.37–8.51)
PRISM III >6, n(%)	71 (31.6)	11 (84.6)	**<0.001**	11.93 (2.58–55.24)
VAP, n(%)	66 (29.3)	9 (69.2)	**0.003**	5.42 (1.61–18.22)
*Enterobacterales*, n(%)	41 (18.2)	4 (30.8)	0.276	2.00 (0.59–6.79)
DR, n(%)	53 (23.6)	4 (30.8)	0.517	1.44 (0.43–4.88)
MDR, n(%)	10 (4.4)	0 (0)	1.000	0 943 (0.913–0.974)
Inotropic, n(%)	78 (34.7)	9 (69.2)	**0.012**	4.24 (1.26–14.21)
VIS>10, n(%)	38 (16.9)	5 (38.5)	**0.049**	3.08 (0.954–9.92)
Days of MV, n (IQR)	9 (6–14)	10 (7–22.5)	0.295	-
ECMO, n (%)	9 (4.0)	2 (15.4%)	0.115	4.36 (0.84–22.67)

Values are expressed as frequency (percentage) for qualitative variables and as median (IQR: Interquartile range) for quantitative variables. PRISM III: Paediatric Risk Mortality III score. VAP: Ventilator-associated pneumonia. DR: Drug-resistant. MDR: Multidrug-resistant. VIS: Vasoactive Inotropic Score. MV: Mechanical ventilation. ECMO: Extracorporeal membrane oxygenation. OD: Odds Ratio. CI: Confidence interval.

## Discussion

To the best of our knowledge, there is no data available comparing the risk factors, outcomes, and microbiological characteristics of severe CAP and VAP in the paediatric population. In this study, we have analysed data from the last five years on children admitted to the PICU with pneumonia, both CAP and VAP. It has revealed some data that is of interest to clinicians, since these are both associated with a high mortality rate, especially VAP, in which mortality can reach up to 20% [28].

The main objective of the present study was to analyse the differences between CAP and VAP in children. According to these results, there are important differences between both diseases, since patients with VAP had worse prognosis than patients with CAP, needing longer mechanical ventilation, more inotropic support and had higher mortality. Moreover, patients with VAP were mainly infected by *Enterobacterales*, while patients with CAP had mainly other gram negative species and gram positives.

The incidence of VAP in our centre during the study was 3.2%, similar to the reported in other studies, in which VAP occurs in 3–10% of ventilated children [29]. Thus, even if we apply routinely a bundle to prevent VAP (elevation of the head of the bed, orotracheal intubation, closed suctioning systems, and daily windows of sedation to evaluate readiness for extubation), VAP is still a major problem in the PICU.

Patients that developed VAP were more prone to having comorbidities than patients with CAP. This is consistent with other studies in adults, in which underlying diseases and comorbidities have been described as risk factors for developing VAP [28]. In addition, patients that developed VAP were more likely to have a tracheostomy than those with CAP. Since tracheostomised children are at an increased risk of developing pneumonia, it is important to implement preventative care bundle measures in these vulnerable patients when they’re admitted to the PICU [30].

Moreover, patients with VAP were admitted due to a cardiovascular disease in the 20% of the cases, which was higher than in patients with CAP. The vulnerability of patients with cardiovascular diseases has been reported in other studies [31], as they are more susceptible to developing VAP and, additionally, nosocomial infections in these patients are an important cause of morbidity and mortality. In some studies performed on adults, they even propose decontamination or pre-emptive antibiotic therapy in order to prevent the development of VAP [32,33].

While patients with CAP were diagnosed with infection at admission, patients that ended up with VAP received this diagnosis later (after 11 days). This fact highlights that even patients that do not develop pneumonia initially are at a high risk of developing it if they are on MV. What is more, according to our results longer length of stay in PICU is an independent risk factor for VAP. It is consistent with the results of other studies, in which length of stay in PICU has been found as a risk factor for VAP [34]. For this reason, strategies to prevent VAP such as the elevation of the head of the bed, regular oral care, maintaining ventilator circuits, the use of cuffed endotracheal tubes, and minimising the duration of MV are highly recommended [35].

Patients with VAP had higher inotropic requirements, longer MV, a longer length of stay, and required more extracorporeal support than patients with CAP. Furthermore, the mortality was higher in patients with VAP than in patients with CAP. However, according to the results of the multivariate analysis, the independent risk factors for death were elevated PRISM-III at admission and the presence of comorbidities. Therefore, patients with VAP had higher mortality probably because that population had more comorbidities and a slightly higher PRISM-III score. Kollef *et al*. described in a recent multicentre study that patients with VAP seem to have worse outcomes than patients with CAP, in terms of mortality rate and length of stay [36]. Therefore, even if both types of patients are in a critical care unit and on MV, we should be especially concerned about the ones with VAP, since they are more likely to require more support and have a poorer outcome.

As for the microbiological data, remarkable differences were found between the two groups. The bacteria most frequently involved with CAP were gram negative species, especially *Haemophilus spp*, followed by gram positive species such as *S*. *pneumoniae* and *S*. *aureus*. In children, CAP is usually caused by a virus, followed by *S*. *pneumoniae* and others like *Haemophilus spp* and *S*. *aureus* [37]. In recent years, the development of vaccines against *S*. *pneumoniae* (the 13-valent pneumococcal conjugate vaccine) and *Haemophilus influenzae type B* has helped to decrease the incidence of CAP related to these microbes, especially severe cases of CAP [21]. Furthermore, after the implementation of these vaccines, other changes in the epidemiology have been revealed: non-vaccine serotypes have been isolated in very few cases but the other hand, an increasing prevalence of CAP with viral involvement has been described [38,39]. Despite this, the cases of CAP related with *S*. *pneumoniae* and *Haemophilus spp* are still relevant, mainly due to persistent inequities in access to healthcare, especially in low and middle-income countries [40]. In this sample, the percentage of CAP due to *S*. *pneumoniae* was 17.2%, which is not negligible. We believe that this could be explained because until 2016 antipneumococcal vaccine was not included in the systematic vaccines calendar, therefore some children were probably not vaccinated against *S*. *pneumoniae* [41]. However, since 2016 the tax of pneumococcal invasive infection has decreased significantly, which is attributable to the systematic vaccination [42]. Currently, 97.9% of the target children for antinpneumococcal vaccine receive it, but only 68% of the adults older than 65 years uptake it [43].

In contrast, VAP was caused mainly by *Enterobacterales*. In adults, it has been widely described how *Enterobacterales* are involved in a high percentage of VAP cases [44]. In our study, the specific microorganisms most frequently involved with VAP were *Pseudomonas aeruginosa*, followed by *Enterobacterales*. In fact, the presence of *Enterobacterales* was found to be an independent risk factor for VAP, which is consistent with the results of previous studies, in which respiratory colonisation, especially due to *Enterobacterales*, has been described to be a risk factor for VAP [45,46].

As previously reported, MV duration and length of PICU stay were significantly longer in the group with VAP. Chomton *et al*. found that the median MV duration at VAP diagnosis was longer for VAP due to nosocomial microorganisms such as *P*. *aeruginosa* or *E*. *coli* when compared with VAP due to community-acquired bacteria such as *H*. *influenzae* and *S*. *pneumoniae* [10]. This fact was also explained by Kollef *et al*.; oropharyngeal and tracheal colonisation with *Pseudomonas* and enteric gram-negative bacilli increases in step with length of hospital stay and severity of illness [47].

One of the major concerns worldwide nowadays is the increasing prevalence of multidrug-resistant microorganisms and the lack of new antimicrobial agents for use in paediatric pneumonia [6]. Patients with VAP required more days of antibiotic treatment than patients with CAP. Moreover, they were more likely to need their antibiotics to be switched. This is probably related to the differences in the type of microorganism the pneumonia involves. While CAP is usually caused by drug-sensitive bacteria and empirical treatment is normally sufficient, in VAP, as reported, there are more MDR and ESBL bacteria, and patients therefore more commonly need broad-spectrum antibiotic treatment. This observation is consistent with the results of other studies, in which ESBL has been related with VAP [48]. In this sample, the length of antibiotic treatment was associated with an increased risk for VAP, but we reckon that probably the relation was that patients with VAP needed longer antibiotic treatment. However, resistant microorganisms are not only a problem in healthcare-associated infections, but also in a community level. In this population, we found that CAP was caused in 22% of the cases by resistant microorganisms; of them, we would like to highlight the presence of MRSA and ESBL, which together caused 5% of CAP. Similar findings have been reported in adults, with 6% of CAP being due to multiresistant bacteria, causing higher mortality [49]. Increasing resistances are one of the major concerns worldwide; concretely, in Spain the 25% of the isolated *S*.*aureus* are MRSA, and around 10% of *Enterobacterales* are ESBL [50]. Some of the factors that are involved in increasing drug resistances are the wide use of antimicrobial agents and the microorganism transmission between humans, and humans and animals. Therefore, urgent measures need to be taken to diminish resistances, since infections caused by resistant microorganisms worsen patients’ outcomes [50].

A significant morbidity and mortality associated with inadequate or delayed antibiotic treatment is reported in adults, so in children it can be assumed that adequate antibiotic use is also a key prognostic factor [10]. In this sample, we did not find differences between patients with CAP and VAP in terms of the appropriate initial antibiotic therapy (87.7% vs. 86.7%, *p* = 0.818), meaning that the microorganism was susceptible to it. We believe that the main reason for not finding differences is that when healthcare-associated infection is suspected, empiric treatment includes coverage for nosocomial microorganisms.

We acknowledge several limitations in this study. Patients admitted to the Neonatal ICU (<1 month) were not included (because the neonatal and paediatric ICUs are two separate units with a high inflow of patients), and therefore we do not have results for the neonatal population. In addition, it is a single-centre study, so the results might be difficult to extrapolate to other populations. However, for a paediatric study, it has quite a large number of patients, so the results may be useful to other PICUs.

Despite these limitations, to our knowledge this is the first study comparing the risk factors and outcomes of severe CAP and VAP in children. Therefore, we believe that it provides valuable information on the paediatric population.

## Conclusions

To sum up, children that develop VAP seem to be more vulnerable than those with CAP, because they had a higher proportion of comorbidities and they had an increased prevalence of cardiovascular diseases. Moreover, patients with VAP required more inotropic support, longer MV, and had longer hospitalisation times than patients with CAP, and they ended up having a higher mortality than patients with CAP. Furthermore, the presence of comorbidities and severity risk score at admission (PRISM III) were independent risk factors related with mortality. In light of this, strategies to prevent nosocomial infections should be carefully executed in order to avoid VAP, since it worsens patients’ prognosis. Finally, considering that VAP is usually related to *Enterobacterales*, and increases with the days of PICU stay, the correct antibiotic treatment should be implemented as soon as signs of infection appear so as to improve the outcome of the patient.
